# Tensile Properties, Collagen Content, and Crosslinks in Connective Tissues of the Immature Knee Joint

**DOI:** 10.1371/journal.pone.0026178

**Published:** 2011-10-13

**Authors:** Sriram V. Eleswarapu, Donald J. Responte, Kyriacos A. Athanasiou

**Affiliations:** 1 Department of Bioengineering, Rice University, Houston, Texas, United States of America; 2 Medical Scientist Training Program, Baylor College of Medicine, Houston, Texas, United States of America; 3 Department of Biomedical Engineering, University of California, Davis, California, United States of America; University of Pittsburgh, United States of America

## Abstract

**Background:**

The major connective tissues of the knee joint act in concert during locomotion to provide joint stability, smooth articulation, shock absorption, and distribution of mechanical stresses. These functions are largely conferred by the intrinsic material properties of the tissues, which are in turn determined by biochemical composition. A thorough understanding of the structure-function relationships of the connective tissues of the knee joint is needed to provide design parameters for efforts in tissue engineering.

**Methodology/Principal Findings:**

The objective of this study was to perform a comprehensive characterization of the tensile properties, collagen content, and pyridinoline crosslink abundance of condylar cartilage, patellar cartilage, medial and lateral menisci, cranial and caudal cruciate ligaments (analogous to anterior and posterior cruciate ligaments in humans, respectively), medial and lateral collateral ligaments, and patellar ligament from immature bovine calves. Tensile stiffness and strength were greatest in the menisci and patellar ligament, and lowest in the hyaline cartilages and cruciate ligaments; these tensile results reflected trends in collagen content. Pyridinoline crosslinks were found in every tissue despite the relative immaturity of the joints, and significant differences were observed among tissues. Notably, for the cruciate ligaments and patellar ligament, crosslink density appeared more important in determining tensile stiffness than collagen content.

**Conclusions/Significance:**

To our knowledge, this study is the first to examine tensile properties, collagen content, and pyridinoline crosslink abundance in a direct head-to-head comparison among all of the major connective tissues of the knee. This is also the first study to report results for pyridinoline crosslink density that suggest its preferential role over collagen in determining tensile stiffness for certain tissues.

## Introduction

**Figure 1:**
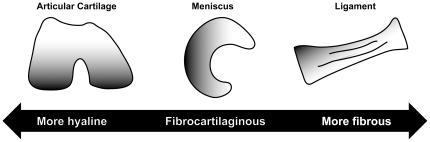
Continuum of knee joint connective tissues. Based on their structural compositions, the major connective tissues of the knee joint can be conceptualized along a continuum from hyaline (condylar and patellar cartilage), to fibrocartilaginous (meniscus), to fibrous (ligament).

The major connective tissues of the knee joint act in concert during locomotion to provide joint stability, smooth articulation, shock absorption, and distribution of mechanical stresses [1]-[3]. These functions are largely conferred by the intrinsic material properties of the tissues, which are in turn determined by their biochemical compositions. Based on structure-function relationships, each connective tissue of the knee joint can be conceptualized along a continuum from hyaline to fibrocartilaginous to fibrous (Figure 1). These tissues have received considerable attention in both basic science and clinical literature, but much work remains to be done to elucidate the contributions of particular biochemical components to important mechanical parameters, especially with respect to applications in tissue engineering. Approaches in tissue engineering are guided heavily by the interplay of native tissue structures and their corresponding functional correlates. To better understand these relationships, this study examines the biochemical composition and tensile properties of the major connective tissues of the immature bovine knee joint.

The knee is a pivotal hinge joint that permits flexion, extension, and limited rotation through coordinated action of its hyaline, fibrocartilaginous, and fibrous connective tissues. Hyaline cartilage is found at the condylar surfaces of the femur and tibia, as well as on the patella. Fibrocartilage comprises the medial and lateral menisci, which are crescent-shaped structures interposed between the femoral and tibial condyles. Fibrous tissue makes up the major ligaments of the knee joint, in particular the patellar ligament, the collateral ligaments, and the cruciate ligaments. The patellar ligament provides stability to the patella as it glides over the patellofemoral groove and femoral condyles. The medial and lateral collateral ligaments (MCL and LCL) are extracapsular ligaments that protect the medial and lateral sides of the knee from a contralateral outside or inside bending force, respectively. The anterior and posterior cruciate ligaments (ACL and PCL) are intracapsular ligaments that stabilize the knee during rotation and bending. Together, these tissues contribute significantly to normal knee function.

The connective tissues of the knee joint are known to derive their mechanical properties from their biochemical components, but precise structure-function relationships remain elusive beyond general notions of the role of the extracellular matrix (ECM). Structurally, each of these tissues is hypocellular and possesses an ECM rich in collagen, with varying amounts of glycosaminoglycans (GAGs) [4], [5]. In general, collagen is known to be largely responsible for the tensile integrity of these tissues, while GAGs, predominant in hyaline cartilage and sparse in fibrous tissues, contribute to compressive strength [6]. In addition to total collagen content, the amount of crosslinking present in the collagen network has been shown to play an important role in tissue tensile properties [7]. In examining tissue tensile properties, two important measures of tensile integrity are Young's modulus and ultimate tensile strength (UTS). Young's modulus is a measure of a material's tensile stiffness, and the UTS is the maximum stress a material can withstand. Though collagen content and crosslinking are known to play a role in tensile mechanics, their precise structure-function relationships with respect to Young's modulus and UTS remain unclear. Pyridinoline crosslinks have been shown to correlate with both tensile strength and stiffness in articular cartilage [8], but there is a dearth of literature describing the contribution of pyridinoline crosslinks to the mechanical behavior of fibrocartilage or ligament tissues.

In humans, conditions afflicting the connective tissues of the knee, such as traumatic injury and osteoarthritis, contribute to substantial healthcare costs and work-related disability [9]–[11]. The field of tissue engineering aims to improve orthopaedic medicine by providing functional replacements for damaged or diseased joint tissues. Recent tissue engineering efforts have focused on major connective tissues such as hyaline cartilage [12], [13], meniscus [14], [15], tendon [16], [17], and ligament [18]. Although various approaches have been employed to engineer these tissues, it has been difficult to reproduce native collagen organization and attain native mechanical properties. Various types of mechanical [13], [19]–[21] and biochemical [22], [23] stimuli have been studied to improve construct properties, and both scaffold-free [12], [24], [25] and scaffold-based [26], [27] approaches have been investigated for connective tissue engineering applications. An additional consideration in these tissue engineering efforts has been the cell source used to produce constructs. Comparisons of cell types have shown that immature cells exhibit increased biosynthesis [28], making them promising candidates for tissue engineering. Immature cells have been used to produce constructs with clinically relevant dimensions [12] and mechanical properties on par with native tissue. To make informed cell source choices, it is necessary to establish a comprehensive understanding of the physiology of immature joint tissues. Moreover, while studies on the knee joint are well represented in the literature, it is important to note that much of what is known about the structure-function relationships of these tissues comes from assessments of adult rather than immature joints, whether human or animal. Given the prevalence of knee injuries in the pediatric population [29], along with a greater push towards using immature tissues as cell sources for tissue engineering, a thorough elucidation of the biochemistry of immature knee joint tissues, not just adult tissues, is warranted. An understanding of immature joint physiology may also yield insight into tissue development by providing a reference to which adult tissues can be compared, as well as informing a general understanding of factors at play in pediatric joint injury. Additionally, because orthopaedic explant and tissue engineering studies are relying more readily on bovine tissues [12], [20], [30]–[32], it is imperative that a full assessment of the bovine joint be undertaken.

The objective of this study was to perform a comprehensive characterization of the tensile properties, collagen content, and pyridinoline crosslink abundance of the major connective tissues of the immature bovine knee joint. Tissues of interest were femoral condylar and patellar cartilage, medial and lateral menisci, cranial and caudal cruciate ligaments (analogous to the ACL and PCL in humans, respectively), medial and lateral collateral ligaments, and patellar ligament. It was hypothesized that trends in tensile properties would reflect those in collagen content; that tensile properties and collagen content would be higher in fibrocartilaginous and ligamentous tissues than in hyaline tissues; and that pyridinoline crosslinks would be found in all tissues, in spite of the immaturity of the tissues. Results from this investigation reinforce the interplay of tissue biomechanics and biochemical content and provide design parameters for future efforts concerned with connective tissue engineering for joint repair.

## Materials and Methods

### Tissue harvest and specimen preparation

Tissue specimens were harvested from the knee joints of 6 one-week-old male bovine calves (Research 87, Boston, MA), shortly after slaughter of the animals for commercial use in the food industry. To normalize variability among animals, each leg came from a different animal. Hyaline femoral condylar cartilage (CC), hyaline patellar cartilage (PC), medial meniscus (MM), lateral meniscus (LM), cranial cruciate ligament (CraCL), caudal cruciate ligament (CauCL), medial collateral ligament (MCL), lateral collateral ligament (LCL), and patellar ligament (PL) were taken. For CC and PC specimens, the cartilage was separated from subchondral bone with a scalpel. For MM and LM specimens, the femoral and tibial surfaces, as well as the inner 1/3 and outer 1/3 portions of the annulus, were sliced away, leaving the approximate interior circumferential portion of the specimen for assessment. CraCL, CauCL, MCL, LCL, and PL were taken whole from their attachments.

From each freshly harvested specimen, a 3 mm dermal biopsy punch was used to obtain samples for histology, quantitative biochemistry, and high performance liquid chromatography (HPLC). The remainder of each specimen was then prepared for tensile testing. Tensile specimens were stored for a maximum of 24 h in phosphate buffered saline with protease inhibitors at 4°C and were allowed to equilibrate to room temperature prior to testing.

### Histology

Samples were cryo-embedded and sectioned at 14 µm. Sections were fixed in formalin for 10 min and then stained with either picrosirius red or safranin O/fast green as described previously [12]. Samples were dehydrated in an ascending series of ethanol and mounted with coverslips prior to imaging.

### Quantitative biochemistry

Biochemistry samples were weighed wet, frozen, lyophilized for 48 h, and then digested in a phosphate buffer with 125 µg/mL papain (Sigma) for 18 h at 65°C. A chloramine-T hydroxyproline assay was employed to quantify total collagen content after 2 N NaOH hydrolysis for 20 min at 110°C [33]. Total collagen was normalized to tissue wet weight and tissue dry weight.

### High performance liquid chromatography (HPLC)

HPLC was performed to quantify the abundance of pyridinoline crosslinks. Samples were weighed wet, digested in 800 µL of 6 N HCl at 100°C for 20 h, and then dried using a vacuum concentrator. Samples were re-suspended in 50 µL of an aqueous solution containing 10 nmol pyridoxine/mL and 2.4 µmol homoarginine/mL and then diluted fivefold with an aqueous solution of 0.5% HFBA acetonitrile in 10% acetonitrile. 10 µL of each sample was injected into a 25 mm C18 column (Shimadzu, Columbia, MD) and eluted using a solvent profile described previously [34]. To quantify the amount of crosslink in each sample, pyridinoline standards (Quidel, San Diego, CA) were employed to create a calibration curve.

### Tensile testing

Each specimen was cut into a dog-bone shape with a 1-mm-long gauge length. Although this tissue preparation may limit comparison to an *in vivo* context, it was important to maintain consistent mechanical testing procedures across tissue types so that comparisons could be made between tissues without the risk of introducing confounding variables. The specimen was photographed alongside a ruler, and ImageJ software was used to determine the width and thickness. A uniaxial electromechanical materials testing system (Instron Model 5565, Canton, MA) was employed to determine tensile properties with a 50 N (CC and PC only) or 5 kN load cell (all other tissues). CC and PC specimens were affixed with cyanoacrylate glue to paper tabs outside of the gauge length for gripping; all other specimens were gripped directly outside of the gauge length. MM and LM specimens were tested in the circumferential direction. CraCL, CauCL, MCL, LCL, and PL specimens were tested in the longitudinal direction. Tensile tests were performed until failure within the gauge length at a strain rate of 1% of the gauge length per second. Force-displacement curves were generated, and stress-strain curves were calculated by normalizing data to specimen dimension. The apparent Young's modulus, a measure of specimen tensile stiffness, was determined by least squares fitting of the linear region of the stress-strain curve. The ultimate tensile strength (UTS) was determined as the maximum stress reached during a test.

### Statistical analysis

All biochemical, HPLC, and tensile assessments were made using *n* = 5–6. To compare among tissues, a single-factor analysis of variance was employed, and a Fisher least significant difference post hoc test was used when warranted. Significance was defined as *p*<0.05.

## Results

### Histology

**Figure 2:**
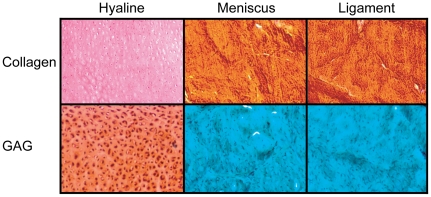
Histology of representative joint tissues. Picrosirius red staining for collagen showed that hyaline cartilage, meniscus, and ligament all had significant collagen content. The meniscus and ligament samples stained more intensely for collagen than hyaline cartilage. Safranin O/fast green staining for GAG showed that hyaline cartilage had significant GAG content; meniscus and ligament did not exhibit GAG staining.

Representative histology for hyaline cartilage, meniscus, and ligament are shown in Figure 2. Staining for collagen was observed in all tissues, though hyaline cartilage exhibited less extensive collagen staining compared to either meniscus or ligament. Extensive staining for GAG was observed in the hyaline cartilage specimens, but was not qualitatively observed in meniscus or ligament specimens.

### Collagen content

**Figure 3:**
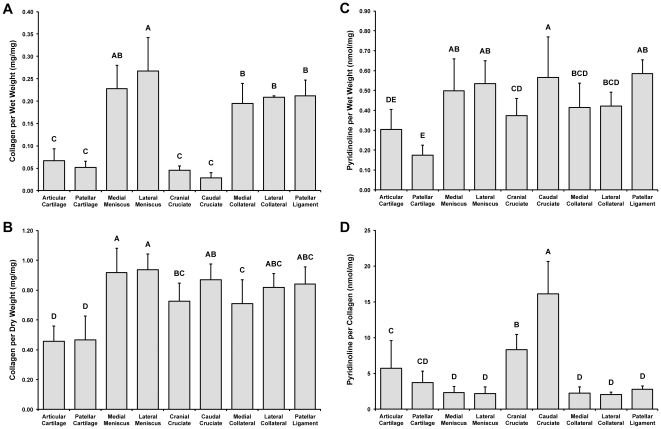
Collagen and pyridinoline content of joint tissues. (A) Collagen normalized to wet weight was significantly higher for the menisci, collateral ligaments, and patellar ligament. (B) Collagen normalized to dry weight was highest in the menisci and lowest in the hyaline cartilages. (C) Pyridinoline normalized to wet weight was highest for menisci, patellar ligament, and the caudal cruciate ligament. Crosslink content was lowest for patellar cartilage. (D) Pyridinoline normalized to collagen was highest for the hyaline cartilages and cruciate ligaments. Groups denoted by different letters are significantly different (*p*<0.05).

The collagen/wet weight for CC, PC, MM, LM, CraCL, CauCL, MCL, LCL, and PL were 6.7±2.6%, 5.1±1.4%, 22.7±5.3%, 26.7±7.5%, 4.6±0.9%, 2.8±1.2%, 19.4±4.6%, 20.9±0.3%, and 21.2±3.5%, respectively (Figure 3A). Fibrocartilage tissues (MM and LM) had the highest collagen content; the fibrocartilage tissues averaged together had 4.1x the collagen content of the hyaline tissues and 6.7x the collagen content of the cruciate ligaments. Among just the fibrous tissues, the collateral ligaments (MCL and LCL) and PL had higher collagen content than the cruciate ligaments (CraCL and CauCL); in particular, the collateral ligaments averaged together had 5.4x the collagen content of the cruciate ligaments. The cruciate ligaments were not significantly different from the hyaline cartilage tissues (CC and PC) in collagen content. The collagen/dry weight for CC, PC, MM, LM, CraCL, CauCL, MCL, LCL, and PL were 45.6±10.3%, 46.5±16.2%, 91.6±16.4%, 93.5±10.6%, 72.6±12.1%, 86.8±10.7%, 70.8±16.1%, 81.7±9.3%, and 84.1±11.6%, respectively (Figure 3B).

### Pyridinoline crosslink content

Pyridinoline was resolved as one peak for all samples. Pyridinoline normalized to tissue wet weight (pyd/ww) for CC, PC, MM, LM, CraCL, CauCL, MCL, LCL, and PL were 0.303±0.101, 0.174±0.049, 0.498±0.160, 0.534±0.115, 0.374±0.087, 0.565±0.204, 0.414±0.123, 0.422±0.067, and 0.585±0.069 nmol/mg, respectively (Figure 3C). Pyd/ww was highest in PL, while CauCL, LM, and MM samples trended higher compared to all other tissues. The hyaline cartilages (CC and PC) had the lowest pyd/ww. The fibrocartilage tissues averaged together had a pyd/ww 2.16x that of the hyaline cartilages, and all of the ligament tissues averaged together had a pyd/ww 1.98x that of the hyaline cartilages.

Pyridinoline normalized to collagen content (pyd/col) for CC, PC, MM, LM, CraCL, CauCL, MCL, LCL, and PL were 5.69±3.85, 3.68±1.59, 2.28±0.88, 2.17±0.92, 8.32±2.12, 16.08±4.53, 2.22±0.85, 2.02±0.34, and 2.80±0.42 nmol/mg, respectively (Figure 3D). Statistically, CauCL had the highest pyd/col and CraCL the second highest, followed by the hyaline cartilages. The collateral and patellar ligaments and both menisci were not statistically different from each other and were less than the cruciate ligaments and the hyaline cartilages. CauCL had a pyd/col 1.93x that of the CraCL, 3.43x that of the hyaline cartilages, 7.22x that of the fibrocartilage tissues, and 7.59x that of the collateral ligaments.

### Tensile properties

**Figure 4:**
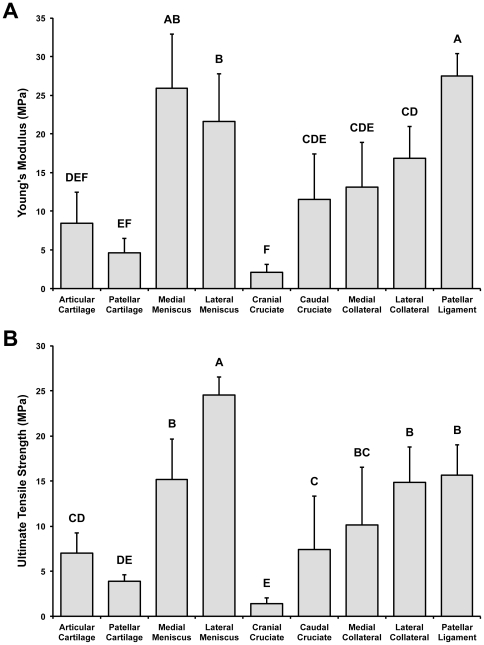
Tensile properties of joint tissues. (a) Young's modulus was highest for the menisci and patellar ligament and lowest for the cranial cruciate ligament. (b) Ultimate tensile strength was also higher for the patellar ligament and the menisci. Groups denoted by different letters are significantly different (*p*<0.05).

The Young's moduli for CC, PC, MM, LM, CraCL, MCL, LCL, and PL were 8.4±4.1, 4.6±1.8, 25.9±7.0, 21.6±6.2, 2.1±1.0, 11.6±5.9, 13.2±5.8, 16.9±4.07, 27.5±2.8 MPa, respectively (Figure 4A). The UTS for CC, PC, MM, LM, CraCL, MCL, LCL, and PL were 7.0±2.2, 3.9±0.7, 15.1±4.5, 24.6±2.0, 1.4±0.6, 7.4±5.9, 10.1±6.4, 14.9±3.9, and 15.7±3.3 MPa, respectively (Figure 4B). MM, LM, and PL exhibited significantly higher stiffnesses (Young's moduli) and strengths (UTS) compared to the other tissues, while CC, PC, CraCL, and CauCL were among the softest and weakest in tensile properties. Also of note, among the cruciate ligaments, CauCL was significantly stiffer and stronger than CraCL; Young's modulus and UTS for CauCL were both 5.4x the values for CraCL.

## Discussion

This study examined the major connective tissues of the immature bovine knee joint, motivated by a need to understand the interplay of biomechanics and biochemistry in immature connective tissues, as well as to establish design parameters for *in vitro* tissue engineering efforts. In the present study, differences were found across tissue types with respect to histology, collagen content, pyridinoline crosslink abundance, and tensile properties. In addition to reinforcing orthopaedic structure-function relationships, to our knowledge, this study is the first to examine these parameters in a direct head-to-head comparison among all of the major connective tissues of the knee, the first to assess pyridinoline crosslink abundance in all the tissues of a bovine joint, and the first to report results for pyridinoline crosslink abundance that suggest its preferential role over collagen in determining stiffness for certain tissues.

In the present study, tissues of interest were first examined histologically for the presence of collagen and GAGs to infer qualitative structural differences underlying the biomechanical distinctions between these different tissues. Meniscus and ligament specimens appeared nearly identical, exhibiting extensive staining for collagen with no observable GAG staining (Figure 2). Hyaline cartilage, by contrast, exhibited less collagen staining than either meniscus or ligament, but also significant GAG staining. These histological trends correspond to the notion of knee joint connective tissues spanning a continuum between hyaline tissue (high collagen, high GAG) and fibrous tissue (high collagen, low GAG) (Figure 1). These qualitative histological differences relate to the functional roles of these tissues: fibrous tissues (ligaments and tendons) and fibrocartilage tissues (menisci) experience tremendous tensile stresses during locomotion, while hyaline cartilage experiences a balance of both tensile and compressive stresses, though preferentially the latter.

Tissue tensile properties, especially in connective tissues, are derived in part from collagen content [6], as well as from other matrix components, such as elastin [35]; therefore, it was hypothesized that trends in tensile properties would reflect trends in collagen content. In this study, collagen content was quantified in each tissue and normalized to tissue wet weight (Figure 3A). It was found that the menisci had the highest collagen content, followed by the patellar ligament and the collateral ligaments. Collagen content was lowest in the hyaline cartilages and the cruciate ligaments. As expected, the tensile properties (Figure 4) appear to reflect the general trends observed in collagen content normalized to wet weight. In particular, it was found that the menisci and patellar ligament exhibited significantly higher stiffness (Young's moduli) and strength (UTS) values compared to the other tissues, while the hyaline cartilages and the cruciate ligaments were among the softest and weakest in tensile properties.

The differences in tensile properties among the ligament tissues (high in patellar ligament, medium in collateral ligaments, and low in cruciate ligaments) may reflect the anatomical development of these tissues, since the stiffer/stronger tissues are extracapsular ligaments, and the softer/weaker tissues are intracapsular ligaments. In particular, the patellar ligament arises from fibers of the quadriceps muscle attaching inferiorly to the tibial tuberosity, hence the term “patellar tendon” often used interchangeably with patellar ligament, given the tendinous origin; the cruciate ligaments develop posteriorly from the articular interzone; and the collateral ligaments form independently of the joint capsule (LCL) or from mesenchymal condensation in the joint capsule (MCL) [36]. Furthermore, of particular interest was the finding that CraCL is significantly softer and weaker than CauCL. Future studies should seek to examine whether this relationship is maintained in adult cows, as well as whether it is observed in humans (i.e., between the ACL and PCL). Taken together, the tensile data described above contribute important information about the tensile properties of immature tissues, especially in light of the increasing incidence of knee joint injuries among youths [29]. Additionally, these tensile properties may serve as important benchmarks to determine success criteria for *in vitro* engineering of the major knee joint connective tissues, all of which play important roles in mechanical function. Tissue engineering efforts aimed at recapitulating native tissue structures should strive to reproduce native tissue biomechanical properties, as well.

Crosslink analysis with HPLC showed that the different joint tissues had varying pyridinoline abundances that contributed to tensile stiffness. The data showed that the hyaline cartilages and the cruciate ligaments exhibited the highest pyridinoline levels (Figure 3). Both the patellar ligament and CauCL exhibited higher tensile stiffness values that paralleled pyridinoline content but not the amount of collagen. Although pyridinoline has been shown to correlate with tensile strength and stiffness in bovine articular cartilage [8], this is the first study to show that pyridinoline also contributes to the mechanical properties of other joint tissues. These results also corroborate structure-function relationships in other species. For example, a study of the rat tendon demonstrated that pyridinoline was a better indicator of ultimate stress than collagen content [37]. These structure-function relationships illustrate the importance of crosslinking in a variety of joint tissues.

Pyridinoline content is known to generally increase as tissues matures, but this study provides comprehensive, quantitative benchmarks that can be compared to adult tissue values. For instance, the observed pyridinoline abundances for condylar cartilage and meniscus fibrocartilage are approximately 50% and 70% of the mature values, respectively [8], [38]. These pyridinoline results can inform future tissue engineering efforts that aim to reproduce the biochemical composition of native tissues. Because engineered cartilage has shown less collagen crosslinking than native tissue, strategies such as increasing lysyl oxidase expression [39] may be needed to increase pyridinoline formation. Other stimuli such as TGF-β1 have been shown to increase pyridinoline content in articular cartilage [40] and could potentially be beneficial for enhancing crosslinking in engineered tissue as well. Considering the role of pyridinoline in tissue mechanics [8], [41] and the inherently mechanical nature of knee joint connective tissues, crosslinking should be a central focus of future tissue engineering approaches.

This study provides biochemical and biomechanical data describing hyaline, fibrocartilaginous, and fibrous tissues of the immature bovine knee joint. These data elucidate important structure-function relationships that can inform directed approaches for functional connective tissue engineering. In particular, future tissue engineering approaches should aim to incorporate methods for improving crosslinking, since crosslink abundance may be a more relevant predictor of tensile stiffness than collagen content for certain tissues, as evidenced by the relationships identified in the cruciate ligaments and patellar ligament. Future work may expand on this study by examining temporal development and maturation of the collagen network and tensile properties, or by making direct comparisons in pyridinoline crosslink abundance between immature and adult tissues. Finally, an assessment of these parameters in disease states such as osteoarthritis or traumatic injury models such as ligament rupture may shed light on predisposing factors.
